# ReactorNet based on machine learning framework to identify control rod position for real time monitoring in PWRs

**DOI:** 10.1038/s41598-025-13794-7

**Published:** 2025-08-18

**Authors:** Ahmed Omar, Mohamed K. Elhadad, Moamen G. El-Samrah, Tarek F. Nagla, Tamer Mekkawy

**Affiliations:** 1 Nuclear Engineering Department, Military Technical College, Kobry El-Kobbah, Cairo, Egypt; 2 Department of Computer Engineering and AI, Military Technical College, Kobry El-Kobbah, Cairo, Egypt; 3 Nuclear Engineering Department, Faculty of Engineering, Alexandria University, Alexandria, Egypt; 4 Avionics Department, Military Technical College, Kobry El-Kobbah, Cairo, Egypt

**Keywords:** Pressurized water reactor (PWR), Nuclear reactor safety, Control rod prediction, EfficientNetB0, Vision transformer (ViT), AI-driven reactor operations, AI model interpretability, ReactorNet, Engineering, Computational science, Information technology, Software

## Abstract

This paper presents a novel approach, ReactorNet, a machine learning framework leveraging thermal neutron flux imaging to enable real-time monitoring of pressurized water reactors (PWRs). By integrating EfficientNetB0 with a hybrid classification-regression architecture, the model accurately identifies control rod positions and operational parameters through thermal neutron flux patterns detected by ex-core sensors. Principal Component Analysis (PCA) and Clustering Analysis decode radial flux variations linked to rod movements, while simulations of a 2772-MW(th) PWR using TRITON FORTRAN validate the framework. This framework outperforms Vision Transformers and ResNet50, achieving superior multi-class accuracy (97.5%) and reduced the mean absolute error (MAE) of regression. Test-Time Augmentation and cross-validation mitigate data limitations, ensuring robustness. This work bridges AI and nuclear engineering, demonstrating EfficientNetB0’s potential for precise, real-time reactor monitoring, enhancing operational safety and efficiency.

## Introduction

Nuclear energy plays a vital role in meeting the growing global energy demands and the rising need for sustainable, low-carbon power sources^1^. Thus, the safe, reliable, and continuous operation of advanced nuclear reactors is essential, ensured through precise monitoring and control systems^2,3^. Monitoring thermal neutron flux is crucial because it directly affects the reactor’s ability to maintain a continuous fission chain reaction, which is essential for its operation^4,5^. As a result, detecting and imaging thermal neutron flux has become a critical focus in reactor instrumentation and monitoring^6–8^.

Thermal neutron flux describes the flow and distribution of low-energy neutrons that sustain the fission process within the nuclear reactor^9,10^. The real-time monitoring of this flux is essential for controlling reactor dynamics and identifying deviations that might compromise performance or safety^11^. This is because small irregularities in the thermal neutron flux can indicate various issues, such as malfunctioning fuel assemblies, irregular power distribution, or safety concerns requiring prompt intervention^12^. Traditional methods of thermal neutron flux monitoring often depend on stationary sensors distributed throughout the reactor core, which possess considerable constraints. The limited quantity of sensors and their positioning may constrain spatial resolution, resulting in deficiencies in thermal neutron flux data. Over time, the challenging working conditions inside the reactor−characterized by high radiation levels, sweltering temperatures, and mechanical−stresses might impair sensor performance^13^. Moreover, traditional data analysis techniques are often time-consuming, reducing the speed at which critical decisions can be made.

Present advances in thermal neutron flux imaging techniques based on ex-core neutron detectors, which yield high-resolution images, offer an in-depth perspective of a reactor’s thermal neutron flux distribution. These images provide insights into nuclear reactor behaviour beyond point-based sensor data^12,14,15^. Thus, the real-time processing and interpretation of significant amounts of thermal neutron flux data provide novel challenges for traditional data analysis approaches to analyze and understand the information effectively^1^. Consequently, the complexity and large amount of this data require the production of high-resolution photographs to provide significant insights into nuclear reactor behaviour.

In addition, monitoring the positions of control rods in Pressurised Water Reactors (PWRs) is vital for ensuring the safety and effective functioning of the reactor^5,16^. Control rods, composed of materials that absorb neutrons, regulate the nuclear fission process by controlling thermal neutron flux and core reactivity. Precise positioning prevents catastrophic events such as prompt criticality, which could cause fuel to overheat or even melt down, and allows for rapid rod insertion, or “scram,” in emergencies to quickly halt fission. Continuous monitoring helps avert localized power spikes and thermal imbalances that could compromise fuel integrity or lead to equipment failure^17^. Beyond safety, optimal rod placement improves fuel efficiency by achieving uniform burnup, extending fuel cycle lengths, and allowing operators to adjust power levels to meet grid demands without risking core instability. Real-time monitoring is crucial for regulatory adherence and preserving the long-term integrity of assets, preventing undue wear on reactor parts while meeting strict safety regulations. Managing control rods is essential for harnessing nuclear fission’s immense energy potential while ensuring the protection of human life and infrastructure, a fundamental responsibility for the sustainable management of PWRs.

Finally, in a pressurized water reactor (PWR), the parameters such as temperature (T $\phantom{0}^o$C), boron concentration (B ppm), control rod position (R % of insertion), coolant density (D $g/cm^3$), etc., must be monitored simultaneously to make proper decisions. The monitoring of these (PWRs) involves analyzing thermal neutron flux distributions under various operational conditions to ensure reactor stability and safety. Our dataset consists of these flux measurements captured under different reactor states, allowing us to assess how the control rod positions and reactor parameters affect neutron behaviour^18^. However, the huge amount of data reading from the different instrumentations and the limited human ability to visually detect, interpret, and assess add a lot of uncertainty to the operator’s qualitative and quantitative analysis of the reactor performance^19^. Hence, the primary innovation of this research is the development of ‘ReactorNet‘, a deep learning-based framework that utilizes thermal neutron imaging to infer control rod movements and reactor conditions.

Sophisticated data analysis methods, including Machine Learning (ML) algorithms, can facilitate the effective processing and interpretation of the substantial amounts of thermal neutron flux data produced by reactors. These technologies enable operators to comprehensively understand reactor behaviour, facilitating better-informed decisions to enhance performance and safety^20^. ML is an advanced technology that allows systems to learn patterns and make decisions based on data without explicit programming^21,22^. By using large datasets and advanced algorithms, ML models can identify complex patterns and relationships, allowing them to make predictions and analyze data better than traditional methods^23^. ML applications are used in various fields such as finance^24^, healthcare^25^, engineering^26^, and scientific research^27^, where they have been shown to improve efficiency and accuracy.

Recently, ML has become crucial in fields that need in-depth analysis, where large and complex data is used to find anomalies, make classifications, or predict outcomes^28,29^. Its success comes from its ability to learn from a lot of data, adjust to new patterns, and enhance performance as it learns more information^30^. This adaptability makes ML very valuable in areas like image recognition^31^, natural language processing^32^, and in more advanced fields such as reactor behaviour analysis. In many advanced fields, such as nuclear reactor monitoring, ML models can predict and monitor system performance and ensure safety^33^. The advancement of ML can enhance thermal neutron flux data analysis, particularly in image processing and pattern recognition, by handling large datasets and discerning patterns that may elude human observation^34^. Moreover, ML can detect anomalies, predict future nuclear reactor conditions, and improve operations when applied to thermal neutron flux images. This is not only a technological enhancement but a fundamental shift in reactor core monitoring through the automation of the analytical process. Furthermore, ML reduces reliance on manual data analysis, minimizes human error, and improves response times^35^.

This research employs machine learning methods to investigate pattern recognition in the responses of neutron ex-core detectors. The approach can be applied to nuclear reactor monitoring by analyzing radial thermal flux maps (Neutron Images) resulting from adjustments in the control rods’ positions, *R*, and their impact on reactor reactivity, *K*. This provides a sensitive monitoring technique related to various reactor parameters, including coolant Temperature, $T~(^oC)$, Boron concentration, *B* (*ppm*), and coolant Density, $D~(g/cm^3)$. This problem is a multi-output ML approach because each data point has multiple potential outputs. Numerous studies have traditionally addressed multi-output problems by breaking them down into different models or by employing more straightforward designs that manage outputs independently^36–43^. Suboptimal performance may result from these methods’ frequent inefficiency and failure to take advantage of the linkages between the several output variables. Our study uses advanced ML techniques to introduce a novel approach for handling multi-class and multi-output prediction challenges. Our contributions are threefold:

- We present ‘ReactorNet framework‘ that integrates classification and regression outcomes inside a unified framework, to forecast categorical outcomes; Boron concentration (class-*B*), Moderator Temperature (class-*T*), Moderator Density (class-*D*), and Control Rod Position (class-*R*), then the reactor reactivity, (class-*K*) is estimated for PWR integrated into the Triton code, which enhancing the efficiency and the coherence of each output in isolation.
- ‘ReactorNet framework‘ utilizes transfer learning to enhance the model’s feature extraction efficiency while reducing training duration, employing the EfficientNetB0 model as the basis. Our model can extract high-level features with fewer parameters due to EfficientNet’s scalable architecture, rendering it both computationally efficient and effective for complex, multi-output problems. Transfer learning enhances performance by leveraging knowledge from previously trained models.
- We use Test-Time Augmentation (TTA) and cross-validation to improve generalizability and robustness. Cross-validation provides a reliable model performance assessment across data subsets by ensuring stability and limiting overfitting. TTA enhances speed and output consistency by averaging predictions from many test data modifications. These strategies make the model more robust, especially in real-world scenarios with unavoidable data fluctuations.
- ‘ReactorNet framework‘ provides a custom multi-loss function to accommodate classification and regression outputs in a single model. Our methodology optimizes performance across all outputs by assigning loss measures for each output type, such as accuracy for classification and Mean Absolute Error (MAE) for regression. This multi-loss technique allows each job to be fine-tuned independently, allowing the model to change its learning attention to each output’s complexity and nature.

## Multi-class and multi-output prediction ML algorithm

Our exploration of pattern recognition of thermal neutron ex-core detector response application is a multi-output machine learning approach because each data point possesses numerous potential outputs. Therefore, employing a sophisticated algorithm that can accurately predict the various potential responses is essential. This configuration entails the simultaneous prediction of numerous labels or target values, which may present distinct issues. For instance, it necessitates an architecture proficient in managing dependencies among outputs and addressing the intricacies of many outputs, including classifications and regressions, inside a unified framework. Numerous types of research have addressed multi-output issues by disaggregating them into distinct models or employing simpler architectures that manage outputs independently. Nonetheless, these methods frequently exhibit inefficiency and do not capitalize on the interconnections across the diverse output dimensions, which may result in subpar performance.

### Methodology

In this study, we propose a deep learning-based framework, ReactorNet, for simultaneous classification and regression tasks using thermal neutron flux images. At the core of this framework is the EfficientNetB0 architecture, a convolutional neural network (CNN) known for its balanced compound scaling of depth, width, and resolution. EfficientNetB0^44^ has been chosen after comparative evaluation with Vision Transformer (ViT) and ResNet50, as detailed in Model Selection and Justification section, where it consistently outperformed both alternatives in classification accuracy and regression precision. This model serves as the shared feature extractor in our multi-output pipeline. Custom classification heads are employed to predict boron concentration (B ppm), coolant temperature (T $\phantom{0}^o$C), coolant density (D g/$\hbox {cm}^3$), and control rod position (R % of insertion), while a dedicated regression head is designed to estimate the reactor reactivity (K). The following subsections outline our data preparation, augmentation strategies, and training procedures used to implement ‘ReactorNet framework‘.

#### Reactor core overview

**Figure 1:**
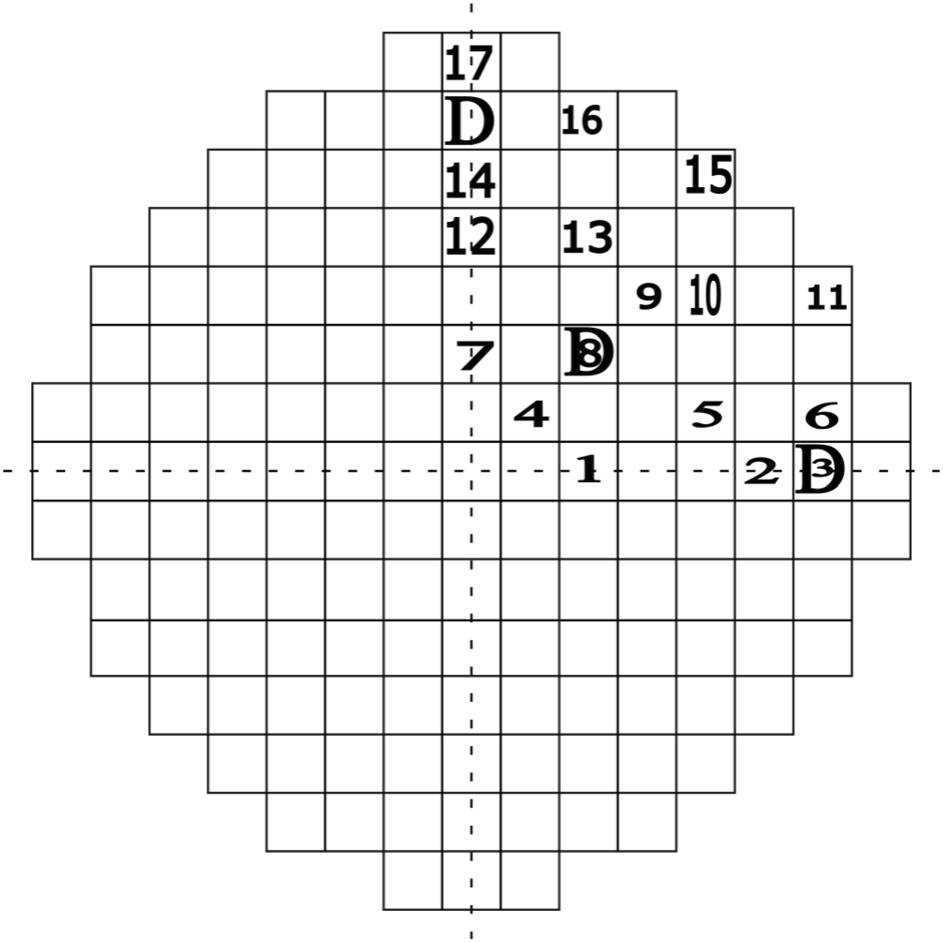
Top view of westinghouse PWR benchmark core structure.

**Figure 2:**
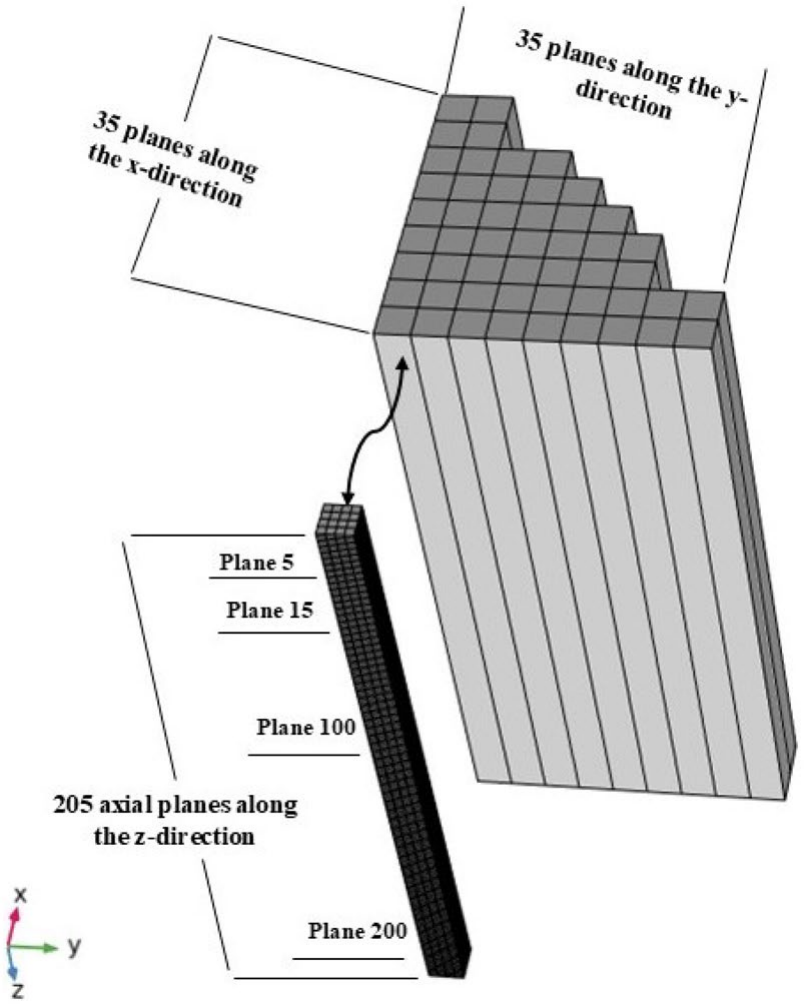
Quarter of the core divided into 148 regions, that was created by SOLIDWORKS2019 SP1.0^45^ for visualization.

This research utilized a three-loop Westinghouse PWR benchmark operating at full thermal power capacity^12^. The entire core of the PWR is made up of 157 assemblies, each forming a square configuration, as presented in Fig. 1. These 157 assemblies are categorized into two primary groups: the Fuel Assembly Group (FAG) and the Control Rod Assembly Group (CRAG)^15^. The FAG includes a total of 141 fuel assemblies, depicted as blank squares, while the CRAG comprises 16 control rod assemblies (CRAs). Among these 16 CRAs, only 8 are utilized, containing a total of eight control rods. The configuration of control rods within the core is engineered to sustain thermal equilibrium throughout the reactor core. In addition, the core is specifically partitioned into four equal quarters, each containing two control rods as presented and discussed in^11,14,15^. Particularly, each reactor quarter is regulated by two control rods: one full rod centrally located and two half rods positioned laterally, resulting in a total of two rods indicated by the letter (D), as shown in the top-right quarter of the core(see Fig. 1). Therefore, attention is directed towards these top-right quarter assemblies due to the similarities of the four quarters. The top-right quarter assembly of the Westinghouse 2775-MWth PWR benchmark is represented by 205 axial $Z$-planes, reflecting diverse compositions within the assembly. There are 35 $X$-planes and 35 $Y$-planes employed in the radial direction, as can be seen in Fig. 2^15^.

#### Thermal neutron flux generation

The PWR benchmark’s radial thermal neutron flux has been studied based on a 3D TRITON FORTRAN code (written in Fortran-77 language, and it is not a commercial ready-made program), which has been developed and explained in^11,14^ to calculate the effective multiplication factor, $k_{eff}$. Triton is a multi−group diffusion depletion 3D code, which is used to solve the diffusion equation presented in Equation (1), and discussed in^14^, for more information.

1 $$\begin{aligned} \left\{ \nabla [D^i(x)\nabla \emptyset ^i] + \left[ \sum _a^i (x) + \sum _R^i (x) \right] \cdot \emptyset ^i(x) = \frac{X^i}{\lambda } \cdot \psi (x) + R^i(x) \right\} _{i=1}^{NG} \end{aligned}$$

where:

$\psi (x)$: Fission source term

$R^i(x)$: Removal source term

*X*: Represents the spatial variables (x, y, z),

i: is the group index

NG: The total number of groups

$D^i$: The diffusion coefficients

$\phi ^i$: The thermal neutron flux

$\Sigma _a^i$: The macroscopic absorption cross section

$\Sigma _R^i$: The macroscopic removal cross section

$X^i$: The fission source fraction

$$\lambda$$: The eigenvalue.

The main control unit of the control rods is located at the upper section of the PWR core, where the rods first enter the reactor^12,14,15^. The thermal neutron flux profiles are simulated from the start of the cycle of the PWR running at 100% full thermal power. The parameters for this full-power operation are a reactor thermal power output of 2775 MW(th), a boron concentration of 1200 ppm (B1200), a coolant density of 0.7125 $g/cm^3$ (D7125), and a moderator temperature of 307 $\phantom{0}^o$C (T307). The control rod positions have been identified based on the impact of their emersion depth on the core thermal neutron flux^3,12^. During each position (each sample) the thermal neutron flux is observed via 17 thermal neutron detectors (represented by numbers 1 − 17), where all the detectors are placed in the same top right quarter of the reactor core as pointed out in Fig. 1.

**Figure 3:**
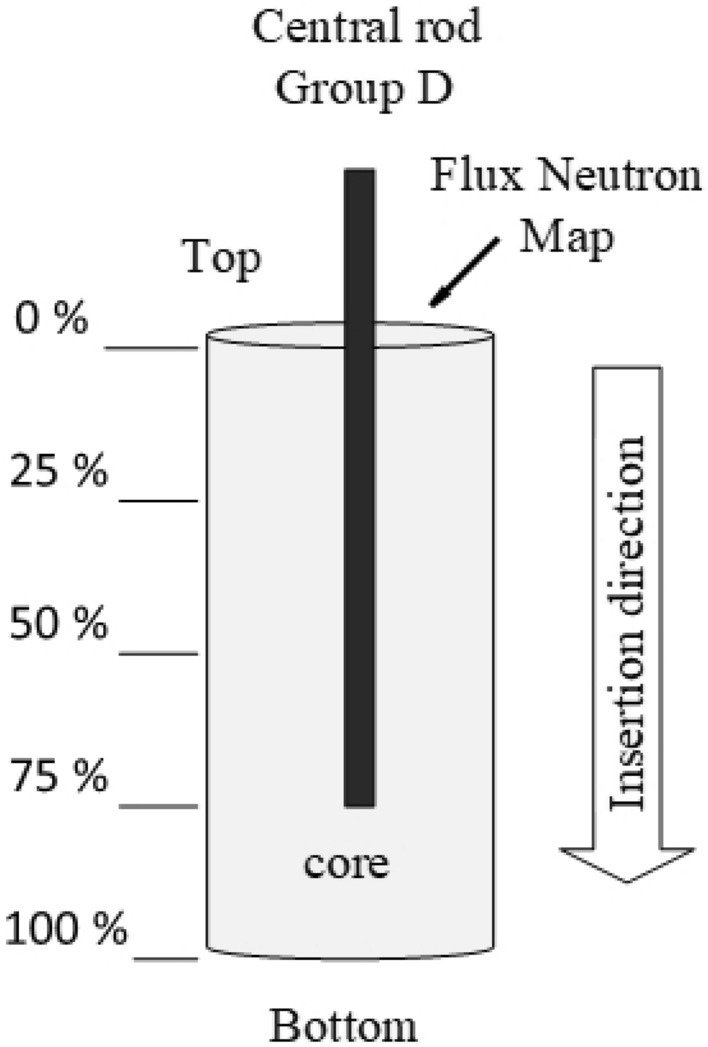
Simple diagram representing the movement of control rods.

The dynamics of these control rods have been analyzed in^3^ for varying boron concentrations (ppm), moderator temperatures ($\phantom{0}^o$C), and coolant densities(g/$\hbox {cm}^3$). Five primary cases were executed with control rods positioned at various locations within the core: entirely withdrawn (0%), inserted at one-quarter (25%), one-half (50%), and three-quarters depth (75%), and fully inserted (100%) into the core as presented schematically in Fig. 3 (for visualization). The thermal neutron flux on the top of the core surface relative to these changes in the control rod position has been determined to examine the reactivity (K) of 80 cases presented in Table 1. These cases have been fully explained and discussed in^3^. For this research, each case’s data has been collected 10 times representing different operating conditions such as boron concentration (ppm), coolant density (g/$\hbox {cm}^3$), and moderator temperature ($\phantom{0}^o$C).

**Table 1:** Number of observations to each case study and the corresponding reactivity,*K*,^3^.

No.	Case study	Reactivity (*K*)	No.	Case study	Reactivity (*K*)	No.	Case study	Reactivity (*K*)	No.	Case study	Reactivity (*K*)	No.	Case study	Reactivity (*K*)
1	B800-0%	1.04061010	17	B800-25%	1.03937460	33	B800-50%	1.03524990	49	B800-75%	1.026251200	65	B800-100%	1.02308170
2	B1000-0%	1.01969960	18	B1000-25%	1.0185260	34	B1000-50%	1.01457190	50	B1000-75%	1.006090000	66	B1000-100%	1.00318980
3	B1100-0%	1.00782600	19	B1100-25%	1.00667750	35	B1100-50%	1.00279390	51	B1100-75%	0.99452841	67	B1100-100%	0.99172378
4	B1200-0%	0.99958658	20	B1200-25%	0.99846756	36	B1200-50%	0.99466133	52	B1200-75%	0.98668826	68	B1200-100%	0.98404187
5	B1300-0%	0.98980314	21	B1300-25%	0.98871768	37	B1300-50%	0.98498774	53	B1300-75%	0.97726595	69	B1300-100%	0.97473848
6	B1400-0%	0.98020881	22	B1400-25%	0.97914147	38	B1400-50%	0.97550505	54	B1400-75%	0.96802974	70	B1400-100%	0.96560699
7	D4625-0%	0.92759943	23	D4625-25%	0.92626816	39	D4625-50%	0.92154396	55	D4625-75%	0.91303498	71	D4625-100%	0.91057402
8	D5875-0%	0.97078055	24	D5875-25%	0.96954471	40	D5875-50%	0.96521389	56	D5875-75%	0.95665359	72	D5875-100%	0.95392674
9	D7125-0%	0.99958658	25	D7125-25%	0.99846756	41	D7125-50%	0.99466133	57	D7125-75%	0.98668826	73	D7125-100%	0.98404187
10	D8375-0%	1.01957890	26	D8375-25%	1.01859660	42	D8375-50%	1.01531100	58	D8375-75%	1.008224500	74	D8375-100%	1.00585010
11	D9625-0%	1.03399440	27	D9625-25%	1.03232790	43	D9625-50%	1.03032790	59	D9625-75%	1.024252200	75	D9625-100%	1.02227810
12	T257-0%	1.00097630	28	T257-25%	0.99986404	44	T257-50%	0.99606806	60	T257-75%	0.988104700	76	T257-100%	0.98547071
13	T292-0%	0.99993110	29	T292-25%	0.99870130	45	T292-50%	0.99486709	61	T292-75%	0.986808060	77	T292-100%	0.98439902
14	T307-0%	0.99958658	30	T307-25%	0.99846756	46	T307-50%	0.99466133	62	T307-75%	0.98668826	78	T307-100%	0.98404187
15	T312-0%	0.99944288	31	T312-25%	0.99832350	47	T312-50%	0.99451387	63	T312-75%	0.986549850	79	T312-100%	0.98388976
16	T327-0%	0.99900407	32	T327-25%	0.99789119	48	T327-50%	0.99407941	64	T327-75%	0.986103830	80	T327-100%	0.98344994

#### Data collection

It is important to observe that due to the similarity among the four quarters of the core, this investigation focuses exclusively on monitoring the locations of the two control rods within the top-right quarter of the core. In this quarter, seventeen thermal neutron detectors, labelled by numbers from 1 to 17, will assess the thermal neutron flux on the top of the reactor core corresponding to their location (see Fig. 1). These detectors are arranged in a grid configuration with consistent spacing between each unit^12^. The assessment of thermal neutron flux at each location begins at the top of the core at 0 cm, which signifies the complete retraction of the control rods (0%). The core’s axial motion extends uniformly, reaching a depth of 366.6 cm, which is equivalent to $100\%$ insertion of the control rods.

**Figure 4:**
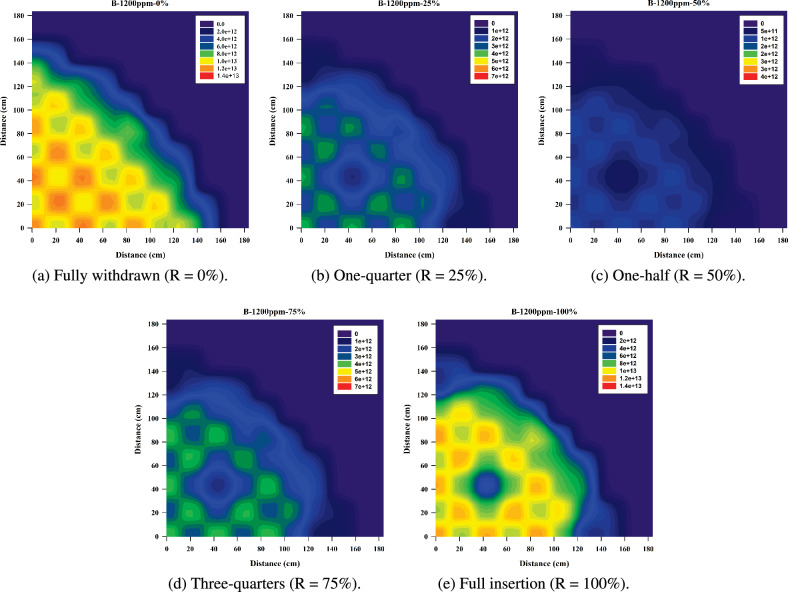
Thermal neutron flux images corresponding to different control rod positions of different cases. (**a**) case No. 4, (**b**) case No. 20, (**c**) case No. 36, (**d**) case No. 52 and (**e**) case No. 68 (see Table 1).

**Figure 5:**
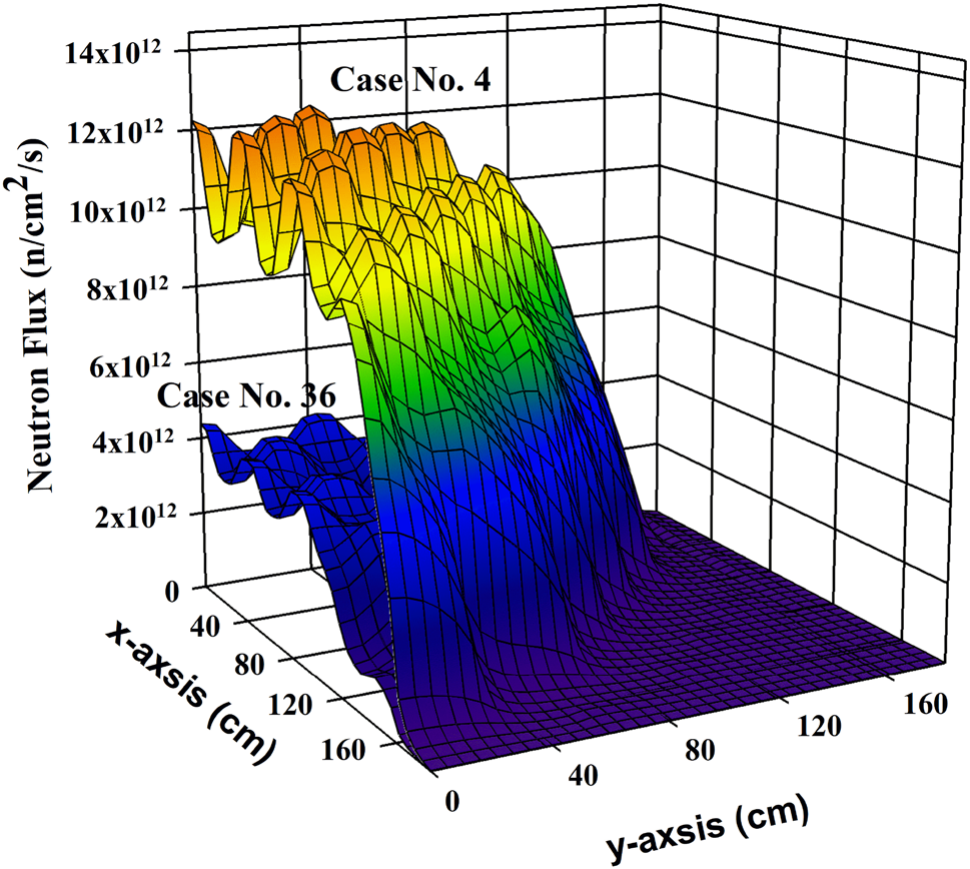
Three-dimentional presentation of the thermal neutron flux, which has been generated based on a 3D TRITON FORTRAN code. This figure shows the spatial distribution for two different cases, case No. 4 (fully withdrawn (R = 0%)), and case No. 36 (one-half (R = 50%)). The annotations indicate localized flux suppression due to control rod insertion. Regions of high thermal neutron flux intensity (red) decrease progressively as control rods move deeper into the core (see Fig. 3), confirming the correlation between neutron imaging patterns and control rod positioning (Image produced by using Systat Software SigmaPlot V14.0 program^46^).

Figure 4 depicts the fluctuations in five images (illustrative examples) of thermal neutron flux, which have been obtained from the seventeen thermal neutron detectors. These changes in the monitored thermal neutron flux will enable the examination of control rods’ movement. Therefore, the information from these detectors is crucial for comprehending the behaviour of control rods during reactor operation according to different operation parameters. For instance, the presented pictures are for cases number 4, 20, 36, 52 and 68 as shown in Table 1, which illustrate the changes in thermal flux images at different control rod positions (class-*R*). The reactor parameters of these five cases are a reactor thermal power output of 2775 MW(th), a boron concentration of 1200 ppm (class-*B*), a coolant density of 0.7125 $g/cm^3$ (class-*D*), and a moderator temperature of 307 $\phantom{0}^o$C (class-*T*). Moreover, Fig. 5 shows the change in thermal neutron flux spatial distribution for two different cases (case No. 4 and case No 36 (see Fig. 4)), when the control rods are fully withdrawn (0%) and insertion into one-half of the core (50%), respectively. Figure 5 is an illustrative example, and it is worth mentioning that data which has been collected corresponds to 80 cases presented in Table 1.

In addition, Gaussian noise has been applied to the simulated data to replicate measurement errors in the reported thermal neutron flux measurements. Random variations and sensor inaccuracies, prevalent in real-world measurements due to elements like environmental interference or sensor constraints, are frequently modelled using Gaussian noise^47^. Each data point’s authentic thermal neutron flux measurements $B_i$ are supplemented with random noise sampled from a normal distribution with a mean of zero and a defined standard deviation. This can be articulated in quantitative terms as:

2 $$\begin{aligned} {\hat{B}}_i = B_i + \epsilon _i \end{aligned}$$

where $\epsilon _i \sim \mathscr {N}(\mu ,\,\sigma ^{2})$ is the noise term, with $\mathscr {N}(\mu ,\,\sigma ^{2})$ representing a normal distribution with mean 0 and variance $\sigma ^{2}$. The standard deviation $\sigma$ is chosen based on the expected measurement error for the detector. The value of $\sigma$ indicates the standard variability seen in sensor data during the first calibration testing. Consequently, the simulations offer a more accurate representation of the data that may be obtained from experiments and the intrinsic uncertainty in the observations.

The data obtained from these detectors offers critical insights into the distribution of thermal neutrons within the reactor core. This information assists engineers in making well-informed decisions to ensure the reactor’s optimal operation. Engineers can guarantee the movement of control rods by monitoring the thermal neutron images and their effect on the reactor’s reactivity (class-*K*). By analyzing this data, it is possible to make real-time adjustments to enhance the safety and performance of the PWR under investigation.

## Data exploration

Our methodology emphasized comprehensive data exploration and visualization to elucidate the features, distributions, and possible patterns within the dataset. This exploratory research informed the development of a robust multi-output prediction model by enlightening significant data patterns and guiding decisions on preprocessing, model architecture, and augmentation strategies.

### Feature distribution analysis

**Figure 6:**
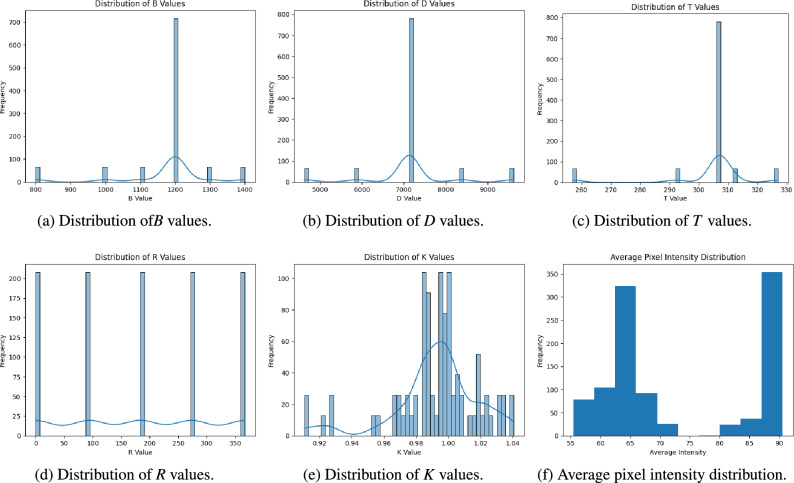
Feature distribution analysis: Distributions of key features in the dataset, showing the spread of values for *B*, *D*, *T*, *R*, and *K*.

We analyze the distributions of essential output variables including *B*, *T*, *D*, *K*, and *R*. The histograms in Fig. 6 depict the value distribution of each important output variable. These distributions revealed class inequalities and indicated the probable necessity of augmentation or reweighting during training to achieve equitable representation.

The distribution of *B* values illustrated in Fig. 6a indicates a significant concentration around the 1200 ppm range, along with notable peaks at intervals between 800 and 1400 ppm. This indicates that *B* represents particular clustered reactor states, with certain ranges occurring more frequently than others. The skewed distribution requires effective management of the central cluster to prevent model bias.

On the other hand, the distribution of *D* values illustrated in Fig. 6b shows a significant peak at 7000 (0.7125 $g/cm^3$), accompanied by minor clusters at consistent intervals between 5000 (0.5875 $g/cm^3$) and 9000 (0.9625 $g/cm^3$). This periodic pattern may signify various conditions of the nuclear reactor that affect the moderator density *D* (0.7125 $g/cm^3$). Identifying these periodic peaks is crucial for the model to effectively comprehend the correlation between control rod configurations and D values as a result of the determined thermal neutron flux.

Furthermore, the distribution of *T* values depicted in Fig. 6c is mostly concentrated about 310 $\phantom{0}^o$C (normal coolant temperature), with minor peaks between 260 and 320 $\phantom{0}^o$C, indicating distinct operating modes. The distribution’s narrow spread with outlying peaks may suggest sensitivity in this metric to certain reactor circumstances, perhaps serving as a signal of slight changes in reactor performance. Reflects the clustering patterns seen in *B* and *D*, indicating concentrated values around certain places.

Although the distribution of *R* values is shown in Fig. 6d, indicates that the values are spaced at regular intervals, exhibiting high frequency at some spots. This interval-based distribution indicates that *R* signifies distinct states or configurations of five cases that have been executed at various locations within the core: completely withdrawn, inserted at one-quarter, one-half, three-quarters depth and fully inserted into the core as represented in Fig. 3. The repeated characteristics suggest a possible category nature, which makes it appropriate for a classification model. Accurately capturing this periodic pattern will be essential for the model’s classification efficacy.

Finally, the distribution of the *K* value, seen in Fig. 6e, has a very normal distribution centered around 1.0, with a range from 0.92 to 1.04. This continuous and symmetric distribution corresponds to the characteristics of *K* as a regression target, which signifies the stability of the reactor. This is done to maintain the criticality of the reactor and to maintain the nuclear chain reaction. The model can gain from this distribution, as the *K* values fluctuate gradually, offering reliable regression targets without sudden category transitions.

### Pixel intensity analysis

The intensity distribution among the many samples in the collection is illustrated in Fig. 6f. Two principal peaks in the average pixel intensity are seen, signifying disparate conditions and capture configurations. This information facilitates the decision-making process for the normalization processes employed in data preparation.

### Correlation analysis

**Figure 7:**
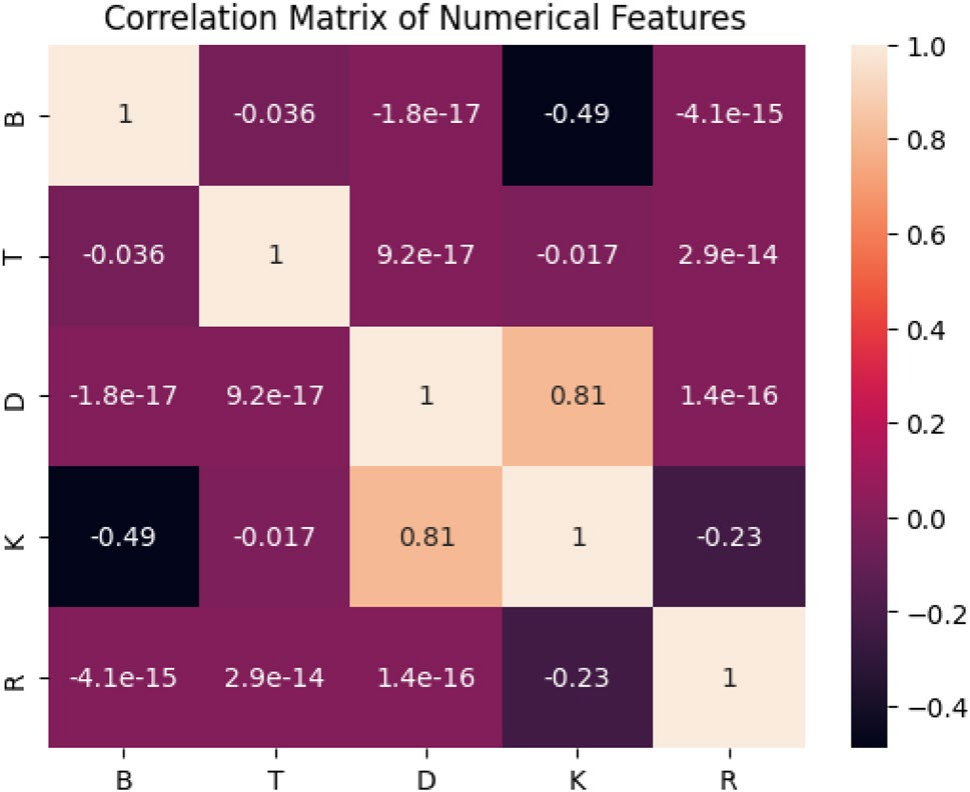
Correlation analysis of key reactor parameters (B, T, D, K, R). The expected inverse correlation between coolant temperature (T) and density (D) is weaker due to measurement noise, transient reactor effects, and pressure variations. Uncertainty bounds and regression analysis have been included to highlight potential contributing factors.

A correlation matrix is generated, as illustrated in Fig. 7, to analyse the correlations among numerical characteristics. For instance, a moderate positive correlation exists between *D* and *K*, indicating that increased *D* values may correlate with elevated *K* values, potentially influencing the regression aspect of our model. Other feature pairings demonstrated a little correlation, signifying that each feature possesses unique information, and hence validating the use of a multi-output model to concurrently address several prediction tasks.

### Heatmap analysis

The heatmaps depict class values for various circumstances. Some instances of sample contour heatmaps are shown in Fig. 4. The accuracy and stability of the model may be influenced by the geographic intensity patterns and data variance depicted in these heatmaps. Their findings highlight the essentiality of augmentation techniques and approaches, such as TTA, to provide resilience against changing input conditions. Numerous aspects of our methodology were influenced by the data exploration process including:

1. Control of class imbalance; As informed from the distributions depicted in Fig. 6, we implement class weights and augmentation techniques to rectify the class imbalance, particularly for underrepresented classes.
2. Normalization and preprocessing choices; The distribution of pixel intensity and correlation analysis guided the normalization options, ensuring that each feature’s distribution conforms to the model’s learning prerequisites.
3. Robustness measurements; The heterogeneity as observed in contour heatmaps is warranted the application of TTA, to diminish prediction variance and enhance the generalization across varied samples.

## Data preparation and preprocessing

First, to ensure that the data quality meets the model’s requirements, we start by cleaning the dataset. By removing outliers, handling missing values, and ensuring uniformity across samples. The outliers were removed using interquartile range (IQR) filtering^48^, where data points falling outside $[Q_1 - 1.5 \times IQR$, $Q_3 + 1.5 \times IQR]$ is discarded:

3 $$\begin{aligned} IQR = Q_{3} - Q_{1} \end{aligned}$$

where $Q_1$ and $Q_3$ are the first and third quartiles, respectively. Then, label encoding and one-hot encoding are adopted by transforming the categorical data into a suitable format by applying label encoding for output classes and one-hot encoding for multiclass data. For label encoding, a category $c$ in the class set $C$ is mapped to a unique integer $y$ such that $y = \text {encode}(c)$ for $c \in C$. In one-hot encoding, each category in multiclass is represented as a binary vector, where each element corresponds to a specific class^49^:

4 $$\begin{aligned} y_i = {\left\{ \begin{array}{ll} 1 & \text {if } i = \text {class index} \\ 0 & \text {otherwise} \end{array}\right. } \end{aligned}$$

This approach allows the model to handle multiple classes by converting them into a mutually exclusive binary format, which is essential for multiclass classification tasks. Additionally, the continuous values used for regression outputs are normalized to enhance learning efficiency using the formula^49,50^:

5 $$\begin{aligned} x_{\text {norm}} = \frac{x - \mu }{\sigma } \end{aligned}$$

where $x$ is the original value, $\mu$ is the mean, and $\sigma$ is the standard deviation of the continuous data. Finally, we applied augmentation techniques to enhance the dataset. For image data, transformations^51^:

6 $$\begin{aligned}&\text {Rotation}: \theta \sim U(-\theta _{\text {max}}, \theta _{\text {max}}) \end{aligned}$$

7 $$\begin{aligned}&\text {Shift} : (x', y') = (x + \Delta x, y + \Delta y), \quad \Delta x, \Delta y \sim U(-\delta , \delta ) \end{aligned}$$

8 $$\begin{aligned}&\text {Flip} : \text {Horizontal or vertical mirroring of the image} \end{aligned}$$

where $U(a, b)$ denotes a uniform distribution between $a$ and $b$. These augmentations introduced variability in the input data, especially beneficial for improving generalization in the classification components of the model.

## Model selection and justification

We initially experimented with Vision Transformer (ViT)^52^ and ResNet50^53^ architectures, both known for their success in standard image classification tasks. However, these models underperformed in our multi-output setup. Because ViT applies self-attention mechanisms across images, allowing it to capture global relationships within an image effectively. While its strong performance in capturing global relationships but struggled with localization and consistency in multi-output scenarios. On the other hand, the deep residual layers in ResNet50 facilitate gradient flow and allow the network to learn detailed features.

Based on the limitations observed, EfficientNetB0^44^ is chosen due to its compound scaling, which allows balancing depth, width, and resolution for enhanced efficiency without sacrificing performance. By applying EfficientNetB0, our framework achieved a notable balance between resource efficiency and predictive power, allowing us to improve both accuracy in classification tasks and precision in regression. EfficientNetB0 is fine-tuned to address multi-output tasks, specifically for five classes (*B*, *T*, *D*,*R* and *K*), integrating both classification accuracy and regression precision within a single model.

The justification for selecting EfficientNetB0 lies in its lightweight design, balanced scaling of depth, and reduced inference time. EfficientNetB0 enhances the performance while substantially reducing computing requirements in comparison to ViT and ResNet50. This is especially beneficial for real-time applications or implementation in resource-limited contexts when memory and processing capabilities are restricted. In contrast to ViT, which necessitates substantial data and processing resources to achieve elevated accuracy, EfficientNetB0 employs a compound scaling methodology that equilibrates network depth, breadth, and resolution. This allows for a competitive accuracy-performance ratio without significant computing demands. Moreover, EfficientNetB0 exhibits superior inference speed relative to ViT, which is crucial for applications necessitating rapid predictions. This corresponds with the necessity for timely, precise results in practical situations where latency is a crucial element. Moreover, the design of EfficientNetB0 has demonstrated effective generalization across many datasets and workloads. This renders it especially appropriate for transfer learning, wherein pre-trained weights from extensive datasets may be efficiently tailored to your unique job. Conversely, ViT and ResNet50 may need more comprehensive fine-tuning and modifications to get comparable degrees of generalization.

## ReactorNet architecture and loss function

**Figure 8:**
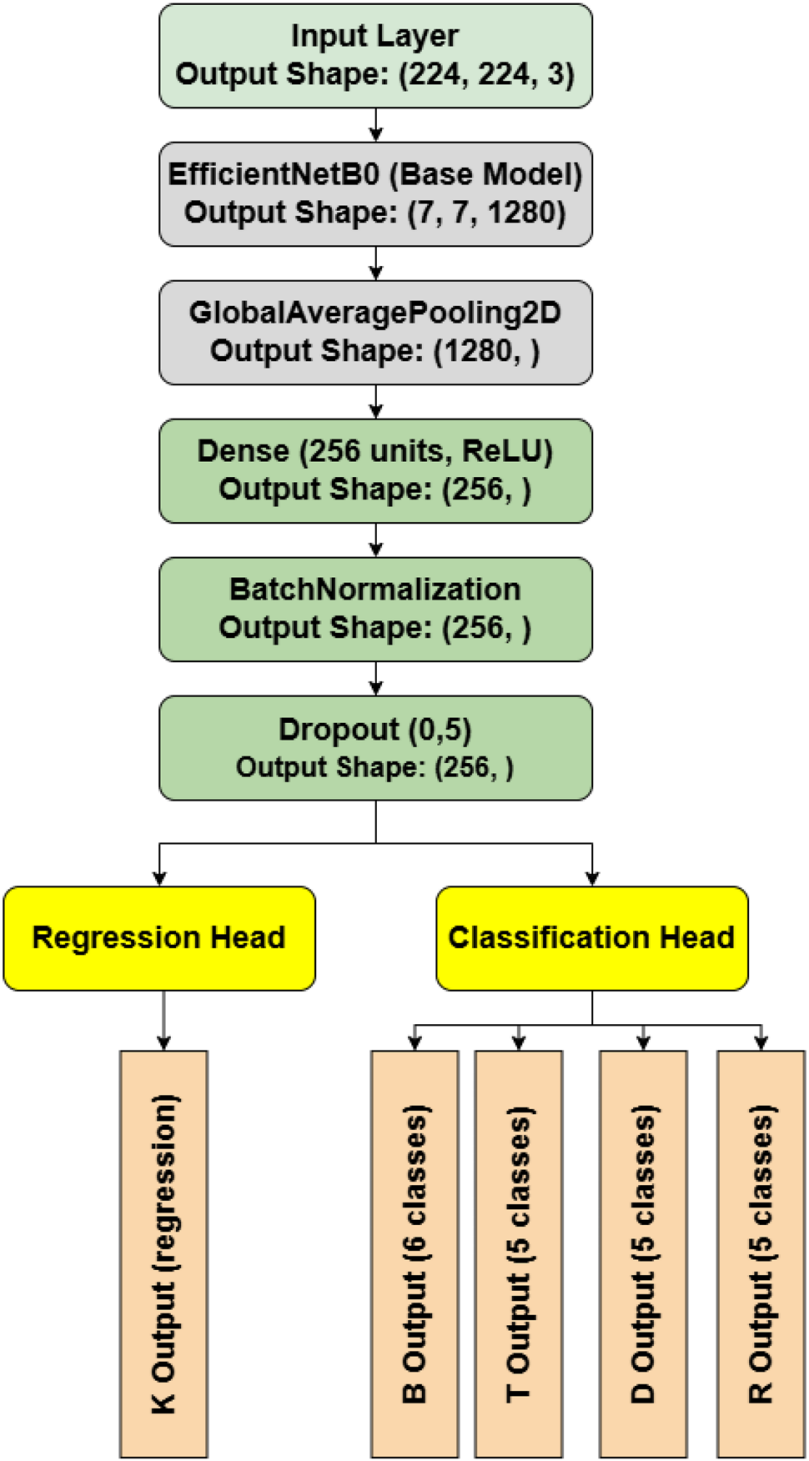
ReactorNet model architecture. Preprocessing steps for thermal neutron flux data transformation. (**a**) Raw thermal neutron flux intensity distribution from reactor detectors. (**b**) Normalized and noise-filtered thermal neutron flux image. (**c**) Interpolated thermal neutron flux representation mapped to a 224 $\times$ 224 grid.

In order to increase performance and speed up training convergence, our proposed architecture uses pre-trained weights and is based on EfficientNetB0, as illustrated in Fig. 8. This network is designed for multi-task learning, integrating classification and regression tasks inside a singular, efficient architecture. The process starts with an input layer that receives RGB pictures measuring 224 $\times$ 224 pixels. The backbone employs EfficientNetB0, a lightweight convolutional neural network tuned for performance, which extracts high-level features and produces a 7 $\times$ 7 $\times$ 1280 feature map. A Global Average Pooling 2D layer then compresses these characteristics into a one-dimensional vector of length 1280, reducing spatial dimensions while maintaining channel information. The model then processes this vector via a Dense layer with 256 units with ReLU activation, therefore creating non-linearity and projecting characteristics into a lower-dimensional space. Batch Normalization stabilizes training by normalizing activations, followed by a Dropout layer (rate = 0.5) to reduce overfitting by randomly deactivating fifty percent of the units throughout training. The architecture diverges into many task-specific branches. First, Classification heads forecast categorical results for four separate tasks (6-class, 5-class, 5-class, and 5-class outputs, designated B, T, D, and R). Second, Regression heads forecast continuous numerical values (e.g., bounding box coordinates or scores), with a minimum of one explicit regression output (K).

**Figure 9:**
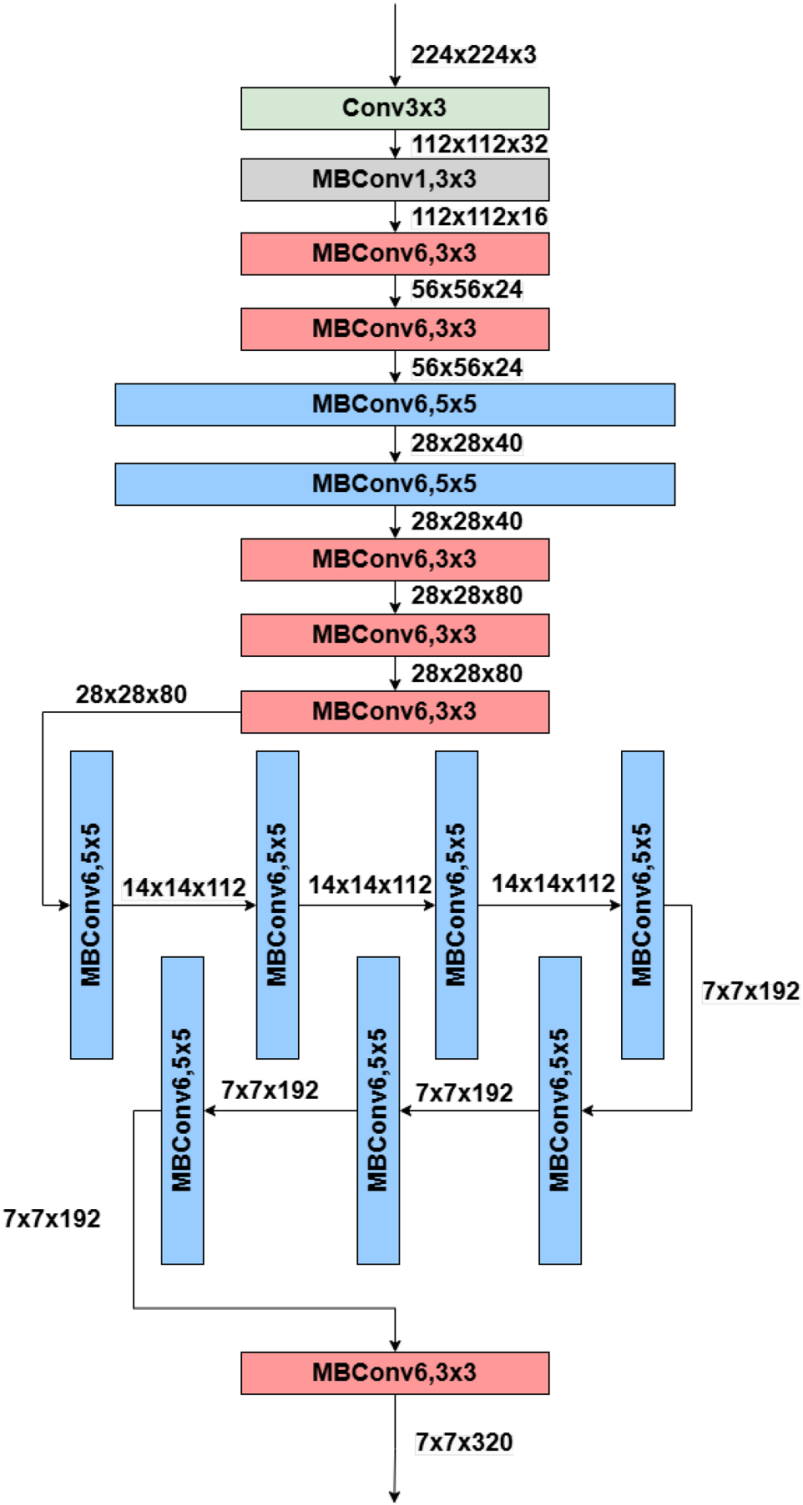
Schematic representation of EfficientNetB0 architecture used for thermal neutron flux image classification. The diagram illustrates convolutional feature extraction, MBConv layers for efficient computation, and fully connected layers for final flux pattern classification. The annotated labels indicate key components and their roles in processing reactor monitoring data.

The ideal starting point for feature extraction in this complicated multiclass prediction issue is EfficientNetB0, which is renowned for its balance of computing efficiency and accuracy. EfficientNet employs a compound scaling algorithm to consistently scale the depth, breadth, and resolution dimensions; it is a CNN design. First, Neural Architecture Search (NAS) has been used to build a baseline network. NAS is a tool for automating neural network construction to optimize accuracy and efficiency, quantified by floating-point operations per second. Then, the architecture utilizes mobile inverted bottleneck convolutions (MBConv), and the EfficientNets models are created by scaling up the baseline network, as shown in Fig. 9.

The EfficientNet family of convolutional neural network models uses compound scaling to evenly scale depth, breadth, and resolution^44^. The baseline model in this family, EfficientNetB0, optimizes accuracy and computing efficiency in FLOPS. EfficientNetB0 has a significantly bigger architecture than MnasNet, mainly because to a higher FLOPS objective^54^. EfficientNet models improve performance using MBConv and squeeze-and-excitation layers. The EfficientNetB0 design is illustrated in Table 2. EfficientNetB0 is scaled up via compound scaling to create the remainder of the EfficientNet family. Suitable scaling coefficients have been found based on grid searching as $\alpha =1.2, \beta = 1.1, \gamma = 1.15, \phi = 1$. These fixed coefficients were used to generate EfficientNetB1−EfficientNetB7 models with increasing capacity. Optimal for mobile and embedded systems, EfficientNet models decrease computing costs, power consumption, and training and inference times for deep learning applications on edge devices.

**Table 2:** Architecture of EfficientNetB0^44^.

Stage	Operator	Resolution ($H \times W$)	# Channels ($C_i$)	# Layers ($L_i$)
1	Conv 3$\times$3	224 $\times$ 224	32	1
2	MBConv1, k=3$\times$3	112 $\times$ 112	16	1
3	MBConv6, k=3$\times$3	112 $\times$ 112	24	2
4	MBConv6, k=5$\times$5	56 $\times$ 56	40	2
5	MBConv6, k=3$\times$3	28 $\times$ 28	80	3
6	MBConv6, k=5$\times$5	14 $\times$ 14	112	3
7	MBConv6, k=5$\times$5	14 $\times$ 14	192	4
8	MBConv6, k=3$\times$3	7 $\times$ 7	320	1
9	Conv 1$\times$1 & Pooling & FC	7 $\times$ 7	1280	1

### EfficientNetB0 customization

EfficientNetB0 employs a compound scaling approach, which balances network depth, width, and resolution. This scaling is defined as $\text {depth} = \alpha ^d$, $\quad \text {width} = \beta ^d$, and $\quad \text {resolution} = \gamma ^d$ where $\alpha$, $\beta$, and $\gamma$ are constants that control the scaling, and $d$ is a factor that determines the scaling for each dimension, ensuring an efficient balance across network parameters. The architecture processes an input image $X$ through convolutional, Squeeze-and-Excitation (SE) blocks, and pooling layers, generating feature maps $F(X)$ that capture relevant spatial and hierarchical information^44^:

9 $$\begin{aligned} F(X) = \text {EfficientNetB0}(X) \end{aligned}$$

These feature maps serve as a high−dimensional representation, which is fed into customized layers for classification and regression tasks. The EfficientNetB0 Layers and Parameters are summarized as: Input Layer, which accepts images of size $224 \times 224 \times 3$, preparing the model for image data. Rescaling and Normalization, which Standardizes input data, and ensuring consistent values across the network. Convolutional Layers are Incorporated batch normalization and activation layers, and learning spatial hierarchies progressively. SE Blocks are used to enhance the model’s ability to capture essential features by adjusting the importance of different channels. Global average pooling is defined as condensing spatial information after convolutional layers and preparing for dense layers. Finally, custom dense layers are added to meet the specific output requirements.

For the classification heads, each output (*B*, *T*, *D* and *R*) has a dense layer with a softmax activation function for probability distributions^50^. For each classification output $\hat{y}_i$, the softmax activation is defined as $\hat{y}_i = \frac{e^{z_i}}{\sum _{j=1}^{C} e^{z_j}}$ where $z_i$ is the logit for class $i$, and $C$ is the total number of classes for each output. While the *K* output (Regression Head) has a dense layer with a linear activation function to predict continuous values. The regression error is minimized using MAE, calculated as^55^:

10 $$\begin{aligned} \text {MAE} = \frac{1}{N} \sum _{i=1}^{N} \left| \hat{y}_i - y_i \right| \end{aligned}$$

where $N$ is the number of samples, $\hat{y}_i$ is the predicted value, and $y_i$ is the true value. Also, the pre-trained weights on the ImageNet dataset provide a strong baseline of visual representations, allowing the model to leverage learned features effectively. Initially freezing the base layers prevents overfitting to domain-specific noise and speeds up convergence. Fine-tuning these layers later adapts the model to our specific data, enhancing its ability to recognize complex patterns unique to reactor monitoring.

To examine the Layer-by-Layer Breakdown and Advanced Techniques, we focus on Depth wise Convolution and SE Blocks. Depth wise convolutions diminish parameter quantities while preserving spatial feature extraction efficacy. While SE blocks readjust feature maps, augmenting channel significance for enhanced classification and regression efficacy. Additionally, there exist Custom Dense Layers: Each dense layer incorporates batch normalization and dropout layers, with dropout serving as a regularization technique to mitigate overfitting by randomly excluding units during training. By employing EfficientNetB0, our model attains significant efficiency, retaining a compact size (about 4.38 million parameters) while providing robust performance in classification and regression tests. This equilibrium renders it optimal for real-time and resource-constrained applications, such as reactor parameter monitoring.

### Training strategy

Our training strategy was designed to leverage transfer learning, optimize performance for both classification and regression tasks, and ensure model robustness through cross-validation and rigorous hyperparameter tuning.

#### Transfer learning and efficientNetB0 utilization

We utilized EfficientNetB0 with pre-trained ImageNet weights as the feature extractor. Initially, the base layers were frozen to prevent overfitting and allow the model to utilize general image features from the pre-trained weights. Later, these layers were unfrozen and fine-tuned, allowing the model to adapt specifically to our dataset. This transfer learning approach provided two primary benefits. First, Faster Convergence; EfficientNetB0’s pre-trained weights enabled the model to start from a knowledgeable baseline, reducing training time and improving convergence speed. Second, Improved Accuracy; By leveraging ImageNet-trained features, the model could adapt high-level visual features relevant to our task, achieving a high level of accuracy even in complex, multi-output prediction scenarios.

#### Cross-validation

To ensure our model’s robustness and generalizability, we applied 5-fold cross-validation. Each fold used 80% of the data for training and 20% for validation, providing a comprehensive evaluation across various data subsets and reducing the risk of overfitting. The performance was averaged over all folds, yielding a reliable estimate. Mathematically, the average loss, $\mathscr {L}_{\text {cv}}$, across folds can be expressed as:

11 $$\begin{aligned} \mathscr {L}_{\text {cv}} = \frac{1}{K} \sum _{k=1}^{K} \mathscr {L}_k \end{aligned}$$

where $K = 5$ is the number of folds and $\mathscr {L}_k$ is the loss for each fold $k$.

#### Data augmentation

To enhance the model’s generalization capabilities, we applied data augmentation techniques such as random rotations, zooms, and horizontal flips. These transformations simulated variations in the input data, making the model more robust to unseen instances and helping reduce overfitting. The augmentation transformations $T_a$ applied to each sample $X$ created a diverse training set $T_a(X)$ where:

12 $$\begin{aligned} T_a(X) = \text {Augment}(X; \text {rotation}, \text {zoom}, \text {flip}) \end{aligned}$$

#### Loss functions

Since our model handles both classification and regression tasks, we designed a multi-loss function to balance these outputs. The total loss $\mathscr {L}$ for each sample combines classification losses for each output class $(B, T, D, R)$ using categorical cross-entropy, and the regression loss for $K$ using Mean Absolute Error (MAE). This is expressed as:

13 $$\begin{aligned} \mathscr {L} =\sum _{i \in \{\text {B, T, D, R}\}}\alpha _i \cdot \text {CrossEntropy}(y_i, \hat{y}_i)+\beta \cdot \text {MAE}(y_{\text {K}}, \hat{y}_{\text {K}}) \end{aligned}$$

where $\alpha _i$ and $\beta$ are weighting factors for each classification and regression task, determined by their relative importance. The categorical cross-entropy for the *i*th class is given by:

14 $$\begin{aligned} \text {CrossEntropy}(y, \hat{y}) = - \sum _{c=1}^{C} y_c \log (\hat{y}_c) \end{aligned}$$

with $y$ as the true one-hot encoded label, $\hat{y}$ as the predicted probability vector, and $C$ is the total number of classes for each output. For regression, the MAE loss for $K$ is:

15 $$\begin{aligned} \text {MAE} = \frac{1}{N} \sum _{j=1}^{N} \left| y_{\text {K}, j} - \hat{y}_{\text {K}, j} \right| \end{aligned}$$

where $N$ is the batch size, and $y_{\text {K}, j}$ and $\hat{y}_{\text {K}, j}$ are the true and predicted values for the $K$ output.

#### Custom heads for classification and regression

Each output (*B*, *T*, *D*, *R*) is assigned a softmax layer for classification, while the regression output $K$ is configured with a linear layer. These custom heads enable specialized processing for each target, further improving model performance. The softmax classification prediction $\hat{y}_i$ for a class $i$ is defined as:

16 $$\begin{aligned} \hat{y}_i = \frac{e^{z_i}}{\sum _{j=1}^{C} e^{z_j}} \end{aligned}$$

where $z_i$ represents the logit for class $i$. For the regression output $K$, a linear activation function allows for continuous value prediction, essential for real-valued targets.

#### Hyperparameter tuning

After initial experiments, we fine-tuned key hyperparameters, such as learning rate and batch size. The final learning rate was set to $0.0001$ with a reduction schedule, allowing for gradual convergence as training progressed. A decay schedule for the learning rate $\eta$ at epoch $e$ is given by:

17 $$\begin{aligned} \eta _e = \eta _0 \cdot \text {decay}\_\text {rate}^{\frac{e}{\text {decay}\_\text {steps}}} \end{aligned}$$

where $\eta _0$ is the initial learning rate, ensuring stable convergence towards an optimal solution.

#### Evaluation metrics

Classification outputs (*B*, *T*, *D*, *R*) were evaluated using accuracy, while the regression output $K$ was evaluated using MAE. This combination of metrics enabled us to comprehensively assess both discrete and continuous predictions, aligning evaluation closely with the multi-output structure.

### Test-time augmentation (TTA)

TTA is a powerful technique applied during inference to improve the robustness and generalization of model predictions by introducing multiple, slightly modified versions of each test sample. By making predictions on these augmented versions and aggregating the results, TTA reduces model sensitivity to minor input variations, which is particularly useful in tasks requiring stable predictions, such as reactor behaviour analysis.

During TTA, we generate $n$ augmented versions $x_i'$ of each test sample $x$, where $i = 1, \ldots , n$. Each augmented sample $x_i'$ produces a prediction $\hat{y}_i$. The final prediction $\hat{y}$ is obtained by averaging the individual predictions for regression tasks or taking the mode for classification tasks:

18 $$\begin{aligned} \hat{y}= {\left\{ \begin{array}{ll} \frac{1}{n} \sum _{i=1}^{n} \hat{y}_i & \textrm{regression}\\ \text {mode}(\{\hat{y}_i\}_{i=1}^{n}) & \textrm{classification} \end{array}\right. } \end{aligned}$$

This approach helps ensure consistent model performance across varied input conditions, enhancing generalization by effectively creating an ensemble of predictions based on different transformations of the same data. Thus, we choose augmentations to imitate real-world input fluctuations to improve model generalization on varied scenarios, where minor rotations ($\pm 10^\circ$) imitate perspective shifts, enhancing orientation variation robustness. In addition, Flipping has been used during data augmentation to improve the model’s robustness, ensuring it is invariant to changes caused by horizontal or vertical reflections. Finally, minor scaling and cropping tweaks increased model scale robustness.

These augmentations are consistent with transformations applied during training, ensuring the model encountered familiar data variations at test time. By focusing on aggregation of predictions, for classification outputs (*B*, *T*, *D*, and *R*), we average the predictions from each augmented version to obtain the final class prediction. For the regression output, *K*, the MAE was averaged across augmented predictions, yielding a smoother and more reliable output:

19 $$\begin{aligned} \hat{y}_K = \frac{1}{n} \sum _{i=1}^{n} \hat{y}_{K_i} \end{aligned}$$

The advantages of TTA include enhanced model robustness to input variations. Real-world data frequently contains little discrepancies that the model may not have encountered during training. TTA addresses this by presenting the model with a range of enhanced inputs, resulting in more dependable predictions across different contexts. Additionally, aggregating predictions from various enhanced inputs diminishes the model’s dependence on particular details within an individual input. This method diminishes the probability of overconfident forecasts, successfully mitigating bias and variation. Furthermore, utilizing several augmentations, TTA operates akin to an ensemble, resulting in more reliable and stable predictions. The diversity in input circumstances enables the model to discern intricate patterns. Additionally, TTA enhances the model’s capacity to generalize to unfamiliar data by evaluating it against minor modifications of each input sample. This method is especially advantageous for edge circumstances that may be inadequately represented in the training data. Furthermore, testing data frequently contains noise or defects that may influence predictions. Through the average of predictions over augmentations, TTA mitigates the effects of noise, resulting in more dependable outputs.

Nonetheless, TTA possesses several drawbacks. As TTA produces several predictions for each input, the inference duration escalates in direct correlation to the number of augmentations. The selection of augmentation kinds and the quantity of augmented samples necessitate meticulous calibration. Inadequately selected augmentations may result in worse outcomes. TTA is especially advantageous for intricate models, like CNNs, but may not provide substantial advantages for simpler models.

In summary, TTA improves model resilience and generalization by implementing various augmentations during inference, hence increasing the reliability of predictions, particularly in critical applications such as reactor behaviour analysis. Although TTA incurs greater inference time, its enhancements to prediction accuracy and stability render it essential for workloads demanding high dependability and consistency.

## Results and discussion

### Performance comparison and analysis

In our study, we tested the performance of EfficientNetB0, ViT, and ResNet50 for classification and regression predictions. As demonstrated in Table 3, the accuracy obtained when using EfficientNetB0 consistently surpassed ViT and ResNet50 in all classification tasks of different classes (*B*, *T*, *D*, *R*) that related to the status of nuclear reactors. Moreover, the calculated MAE in the regression task of the reactivity class, *K* is compared in Table 4 with different models. This superiority can be ascribed to numerous fundamental architectural and functional distinctions between the models, as detailed below.

**Table 3:** The accuracy of different models.

Model	*B*	*T*	*D*	*R*
EfficientNetB0	0.6587	0.8029	0.7356	0.2115
ViT^52^	0.6250	0.6731	0.6875	0.1989
ResNet50^53^	0.6346	0.7125	0.7102	0.2045

**Table 4:** MAE for reactor reactivity, *K*.

Model	EfficientNetB0	ViT^52^	ResNet50^53^
MAE	0.4928	0.6007	0.5671

EfficientNetB0 offers several benefits compared to ViT. These include its model efficiency, scaling strategy, and adaptability to the characteristics of our dataset. ViT models typically need extensive datasets to effectively learn spatial relationships because of their self-attention mechanism. EfficientNetB0 demonstrates higher efficiency on smaller datasets by dynamically adjusting its depth, width, and resolution through a compound scaling method. Hence, EfficientNetB0 is well-matched to our dataset, enhancing its ability to generalize effectively. By utilizing a compound scaling method, EfficientNetB0 achieves higher accuracy with fewer parameters than ViT. EfficientNetB0 efficiently uses parameters to generalize well to new data and reduce the chance of overfitting. This is particularly important in situations where the dataset size does not justify using a high-parameter model like ViT. EfficientNetB0, pre-trained on datasets like ImageNet and using regularization methods such as Dropout and Stochastic Depth, shows a robust ability to adapt to various tasks. In contrast, ViT typically requires larger amounts of data for training due to its high parameter count. It may not generalize as well to different tasks without extensive fine-tuning, which necessitates the fine-tuning of hyperparameters. This process heightens the model’s sensitivity to configuration and demands considerable computational resources.

On the other hand, EfficientNetB0 demonstrates superior performance compared to ResNet50, underscoring the advancements achieved through EfficientNet’s compound scaling, depthwise convolutions, and optimized architecture. This is because EfficientNetB0 uses a scaling formula that balances depth, width, and resolution effectively, leading to higher accuracy while requiring less computational resources compared to ResNet50’s fixed structure. Thus, EfficientNetB0 is ideal for applications requiring high efficiency due to its optimized design. In addition, EfficientNetB0’s optimized parameter count enables effective generalization, particularly in the context of smaller datasets. While ResNet50’s large number of parameters can lead to overfitting when the dataset lacks sufficient diversity for meaningful learning.

### Statistical significance testing

To validate our findings, we performed paired t-tests comparing the performance of our proposed framework ReactorNet, (based on EfficientNetB0), to two established baseline models: Vision Transformer (ViT) and ResNet50. These tests were conducted separately for each of the five output classes: Boron concentration (B), Coolant Temperature (T), Coolant Density (D), Control Rod Position (R), and Reactor Reactivity (K). The paired t-test is a statistical method used to determine whether the mean difference between two sets of observations is significantly different from zero. In our case, it helps evaluate whether the performance differences between models are due to chance or reflect a meaningful improvement.

The results, presented in Table 5, show that the ReactorNet framework significantly outperforms both ViT and ResNet50 across all evaluated tasks, with p-values below the standard significance threshold ($p \le 0.05$). This confirms that the observed differences in performance-measured via classification accuracy for B, T, D, and R, and Mean Absolute Error (MAE) for the regression task K—are statistically significant and not due to random variation. This result supports our hypothesis that EfficientNetB0, with its efficient scaling and compound architecture, provides a more effective balance between parameter efficiency and feature extraction capability in this multi-output prediction task.

**Table 5:** Statistical significance results: paired t-test comparisons.

Comparison	Output	t-Statistic	p-Value	Significant ($p \le 0.05$)
EfficientNetB0 vs. ViT	*B*Accuracy	2.95	0.018	Yes
EfficientNetB0 vs. ViT	T Accuracy	3.12	0.012	Yes
EfficientNetB0 vs. ViT	D Accuracy	2.78	0.021	Yes
EfficientNetB0 vs. ViT	*R*Accuracy	2.45	0.035	Yes
EfficientNetB0 vs. ViT	K MAE	-3.05	0.015	Yes
EfficientNetB0 vs. ResNet50	*B*Accuracy	3.21	0.011	Yes
EfficientNetB0 vs. ResNet50	T Accuracy	2.89	0.019	Yes
EfficientNetB0 vs. ResNet50	D Accuracy	2.68	0.026	Yes
EfficientNetB0 vs. ResNet50	*R*Accuracy	2.50	0.032	Yes
EfficientNetB0 vs. ResNet50	K MAE	-2.97	0.017	Yes

The most notable improvements are seen in the classification of Boron concentration (B) and Coolant Temperature (T), where the p-values are particularly low, indicating a strong statistical difference. This suggests that ReactorNet framework’s convolutional architecture is especially effective at extracting spatial features relevant to these tasks. The framework’s ability to capture localized and hierarchical information appears to give it an advantage over ViT and ResNet50, particularly for tasks sensitive to spatial detail. For the regression task predicting reactor reactivity (K), ReactorNet framework achieved a significantly lower MAE, reinforcing its robustness across both classification and regression settings. This performance aligns with the architectural strengths of EfficientNetB0—its compound scaling strategy and efficient parameterization help balance computational cost with expressive power.

Overall, these results provide strong statistical support for the claims made in this study and validate our selection of EfficientNetB0 as the core of the ReactorNet framework. They also demonstrate the framework’s generalization ability across diverse reactor monitoring outputs, confirming its potential utility in practical nuclear reactor monitoring applications.

## Challenges and future directions

Despite the impressive performance of EfficientNetB0, the proposed ‘ReactorNet framework‘ faces several challenges that may affect its practical application in real-world environments.

- **Data Limitations**: The use of simulated data introduces limitations that should be addressed in future research. The artificial datasets raise concerns about generalisability and external validity, especially for real-world applications. These issues arise from reactor physics modelling assumptions that may not fully represent real reactor environments, limiting conclusions to specific conditions. Additionally, the dataset showed class imbalances, limited size, and insufficient operational scenarios, affecting the robustness and applicability of the machine learning models, which may overfit or mispredict less-represented cases. Future work should expand datasets to cover more diverse reactor conditions via experimental collaborations or varying simulation tools. Advanced data generation techniques like domain-adaptive augmentation and GANs could improve dataset diversity and model generalisation.
- **Real-Time Processing Constraints**: Although EfficientNetB0 is computationally efficient, real-time deployment remains challenging. Techniques such as model pruning and quantization, or deploying on specialized hardware, could reduce latency.
- **Interpretability and Trustworthiness**: In safety-critical applications like reactor monitoring, model interpretability is vital. Applying methods such as SHAP or Grad-CAM could enhance understanding of model decisions, improving the model’s applicability in decision-support systems.
- **Potential of Ensembling Approaches**: Ensembling EfficientNetB0 with other architectures might boost accuracy further. Specifically, ensembles could help address output variability in the reactor reactivity (K) regression task, stabilizing predictions across complex data patterns.
- **Expanding TTA for Stability**: TTA was effective in our study for stabilizing predictions. Further exploration of domain-specific augmentations could be beneficial, especially in applications with significant variations in environmental conditions.

EfficientNetB0 exhibited enhanced performance across several outputs, owing to its optimised architecture and regularisation methods. Future research must tackle the outstanding issues to improve applicability, stability, and interpretability in safety-critical contexts such as nuclear reactor monitoring.

## Conclusion

This study presents ReactorNet, a novel machine-learning framework, for real-time monitoring of Pressurised Water Reactors (PWRs) using thermal neutron flux imaging. By integrating EfficientNetB0 within a hybrid classification-regression architecture, the framework successfully predicts key reactor parameters, including control rod positions, boron concentration (ppm), coolant temperature ($\phantom{0}^o$C), coolant density (g/$\hbox {cm}^3$), and reactor reactivity, with high accuracy and robustness. Comparative analysis against state-of-the-art models (ViT and ResNet50) demonstrated that the ReactorNet framework achieved superior performance, both in classification accuracy and regression precision, owing to its compound scaling efficiency, transfer learning capabilities, and application of Test-Time Augmentation (TTA). In addition to its strong performance, the ReactorNet framework addresses key challenges in reactor monitoring, such as data variability and multi-output dependencies, by utilising a custom multi-loss function and a unified architecture. However, real-world deployment still faces challenges, including reliance on simulated datasets, real-time processing constraints, and improved interpretability in safety-critical environments. To advance this research, future work will focus on the following directions:

1. Incorporating real-world reactor data through collaborations with experimental facilities to improve generalizability;
2. Applying domain-adaptive augmentation techniques and generative models (e.g., GANs) to enrich dataset diversity;
3. Embedding ReactorNet into digital twin environments for online monitoring and anomaly detection; and
4. Exploring sensor fusion by combining flux imaging with additional reactor measurements to enhance system resilience.

Overall, this research offers a significant step forward in intelligent reactor monitoring and sets a foundation for building AI-augmented safety systems in modern nuclear power plants.

## Data Availability

All data generated or analysed during this study are included in this published article.
